# Neurocognitive Functions Among Patients Dependent on Natural Opium: A Comparative Cross-Sectional Study

**DOI:** 10.7759/cureus.84621

**Published:** 2025-05-22

**Authors:** Raghvendra S Singh, Navratan Suthar, Mukesh K Swami, Tanu Gupta, Naresh Nebhinani, Dharamveer Yadav

**Affiliations:** 1 Psychiatry, All India Institute of Medical Sciences, Jodhpur, Jodhpur, IND; 2 Clinical Laboratory/Medicine Biochemistry, All India Institute of Medical Sciences, Jodhpur, Jodhpur, IND

**Keywords:** cognitive dysfunctions, executive functions, natural opioids, neurocognitive functions, opium, working memory

## Abstract

Introduction: Opioid use has been associated with neurocognitive impairments. These deficiencies play a key role in perpetuating addictive behaviors and hindering the effectiveness of motivational and cognitive treatments. Research on the cognitive effects of natural opium use is limited. This study aims to compare the neurocognitive functions of patients dependent on natural opium with those of healthy controls and examine the association of cognitive functions with clinical variables among cases.

Methods: This cross-sectional study involved 26 patients dependent on natural opium and 26 healthy controls matched for age and gender. A neuropsychological test battery, including the Digit Symbol Substitution Test (DSST), Stroop Color-Word Test (SCWT), F-A-S Test, Digit Span Test (DST), Trail Making Test (TMT) A & B, and Rey Auditory Verbal Learning Test (RAVLT), was used for assessment. Urinary opioid levels were measured in the patients. Partial correlation was employed to investigate the association of cognitive functions with the level of exposure to natural opium.

Results: The demographic profiles were comparable, with mean ages of 36 and 33 years for cases and controls, respectively. Cases performed significantly worse on the Stroop test (color word (t = -4.564, p < 0.001), interference scores (t = 2.304, p = 0.025)), the Digit Span Test (domain of sequencing (U = 479, p = 0.005)), the Trail Making Test (part B duration (t = 3.631, p = 0.001)), and the RAVLT (errors of hit (t = -3.119, p = 0.003) and omission (t = 2.990, p = 0.004)). In partial correlation analysis, the duration of opioid dependence was significantly correlated (p < 0.05) with Stroop interference (-0.481) and DST sequence (-0.464). Urine opioid levels were markedly correlated with Stroop CW (0.533).

Conclusion: Response inhibition, cognitive flexibility, and working memory were the most affected domains of cognitive functioning, suggesting that cognitive dysfunction is limited and not global among natural opium users. The urinary opioid levels and the duration of opioid dependence showed a significant correlation with cognitive impairment. However, these effects were exploratory and cannot be generalized.

## Introduction

In Western Rajasthan, India, natural opioids (opium) frequently serve as a ceremonial beverage and are woven into the local community's sociocultural fabric. They are used in two forms: the resinous form (Amal) and the dry husk (Doda). Opium is also employed for self-medication to manage physical and emotional discomfort [1]. During routine clinical assessment, users perceive more benefits than harms, reporting that it enhances their physical and mental work performance.

Impaired cognitive modulation of downstream motivational processes, whether aversive (stress, negative affect) or appetitive (reward, incentive salience), is a crucial factor in addictive disorders, making it a potentially significant target for intervention. Memory, attention, executive functions, and decision-making (including reward expectation, valuation, and learning) are all moderately impaired in individuals with substance use disorders (SUDs), and deficiencies in higher-order executive functions and decision-making serve as important predictors of relapse [2].

Chronic opioid use has been linked to abnormalities in various cognitive domains, particularly executive functions. However, cognitive impulsivity (risk-taking), verbal working memory, and cognitive flexibility have shown substantial impairment [3]. Furthermore, it has been reported that the extent and severity of cognitive impairment are particularly high in individuals with active opioid dependence who are using potent synthetic opioids compared to those using pharmaceutical opioids (Buprenorphine and methadone); limited research has suggested that it is relatively spared among patients dependent on natural opioids [4].

Learning new skills to prevent relapse, controlling impulses and automatic thoughts, and creating problem-solving strategies are all necessary for adopting a new lifestyle and maintaining abstinence. Cognitive dysfunctions lead to poor treatment adherence and lower self-efficacy, resulting in shorter durations of abstinence than expected. This issue is not uncommon among opioid users, even those with mild cognitive impairments [5]. An individual's ability to benefit from therapy may be hindered by such cognitive deficiencies, necessitating extensive additional efforts to assimilate strategies for sustaining abstinence [6]. These patients may have specific treatment needs and benefit from including cognitive-enhancing approaches within the SUD treatment intervention. Consequently, researching individuals' neurocognitive profiles may be crucial in influencing therapeutic decisions.

However, the existing literature is limited because this research primarily focuses on individuals consuming mixed substances, including mixed opioids, synthetic opioids, or pharmaceutical opioids. It lacks an assessment of cognitive functions in a withdrawal-free state, a comprehensive evaluation of cognitive functions using a wider range of neuropsychological tests, and the quantification of opioid dosage through urine analysis.

Considering that natural opium is often culturally accepted with perceived benefits that outweigh the harms, along with concerns about treatment reluctance and higher relapse rates, it is crucial to gather data on its effects on cognitive functions. This research aims to raise awareness among the general public and patients, as well as to design interventions targeting specific cognitive domains that may impede treatment.

The hypothesis was that natural opium consumption could not impair neurocognitive functions. Therefore, the current study was conducted with the primary objective of assessing and comparing the cognitive functions of patients dependent on natural opium with age- and gender-matched healthy controls. In addition, the study examined the association of cognitive functions with clinical variables, including urine opioids, among patients dependent on natural opium as a secondary objective.

## Materials and methods

After receiving approval from the Institutional Ethics Committee (certificate reference number: AIIMS/IEC/2020/3138), a comparative cross-sectional study was conducted at All India Institute of Medical Sciences, Jodhpur, a tertiary care hospital in northwestern India, from February 2021 to February 2022. The study included two groups: male patients with opioid dependence (dependent on natural opium), as defined by the ICD-11 criteria, who were selected as cases, and healthy subjects, matched by age and gender, who served as controls.

All treatment-naïve male patients dependent on natural opium, aged 20 to 50 years, who could read, write, and understand either Hindi or English, were included in the study as cases. Patients with a history of chronic medical disorders, epilepsy, head injuries, neurological disorders, the presence of other substance dependence, except for nicotine, comorbid psychiatric disorders, and an IQ below 80 (as tested using the Wechsler Adult Intelligence Scale-4), as well as those with color blindness, were excluded. Age- and gender-matched healthy subjects without a history of substance dependence, except for nicotine, psychiatric illness, or chronic medical/neurological conditions, were selected as controls from caregivers or attendants of other patients. Females were excluded due to minimal presentation in a hospital setting.

The available literature indicates that verbal memory and executive functions are consistently impaired in patients with opioid dependence [3,7]. The sample size was calculated using an online calculator (openepi.com) based on findings from the Trail Making Test and the Rey Auditory Verbal Learning Test (RAVLT) in a previous study, maintaining a two-tailed significance level of 0.05 and 80% power [8]. The final calculated sample size was 26 for each group. However, this is a small sample size to generalize the findings. Patients were enrolled using a non-probability convenience sampling method. For each patient, a healthy control matched for age and education was recruited through word of mouth from the patients' bystanders.

The participants who met the selection criteria were recruited after obtaining written informed consent. The sociodemographic and clinical profiles (age of onset, duration of use, duration of dependence, form and amount of opioid use, previous attempts at abstinence, family history of psychiatric illness and substance abuse, and withdrawal scores at baseline and before cognitive assessment, etc.) of the patients were recorded. Quantitative urine analysis for opium was conducted at the time of assessment using a commercially available Human Opioid Peptide (OP) ELISA kit based on the Sandwich-ELISA method. Its sensitivity is 0.123 ng/ml, with a detection range of 0.32-20 ng/ml.

The severity of opioid dependence was assessed using the Severity of Opioid Dependence Questionnaire (SODQ), a nine-item self-administered questionnaire divided into five sections: quantity and pattern of opiate use (usual route of administration, etc.); physical symptoms of withdrawal (symptoms upon awakening before the first dosage); and mood symptoms related to withdrawal, including cravings and mood states upon waking before taking the first dose, withdrawal-relief drug use, and the reinstatement timeframe of withdrawal symptoms following a period of abstinence. It has 21 questions with a four-point Likert scale from 0 to 3, with a score range from 0 to 63. [9]. The Fagerström Test for Nicotine Dependence (FTND and FTND-ST) was employed to assess the degree of nicotine addiction. Both questionnaires have six questions. Multiple-choice questions are marked from 0 to 3, whereas yes/no questions are rated 0 or 1. The items are added to create a final score between 0 and 10. The more severe the patient's physical reliance on nicotine, the higher their overall Fagerström score is [10]. In addition, a cognitive assessment was conducted if the patient had a withdrawal score <5 based on the Clinical Opioid Withdrawal Scale (COWS). It is an 11-item assessment tool used to measure the severity of opioid withdrawal symptoms in patients and total scores are grouped as "mild" (five to 12 points), "moderate" (13 to 24), "moderately severe" (25 to 36), and “severe" (more than 36) [11]. These assessments were performed during the first contact when participants had consumed their morning dose of natural opioids and had not yet started any medication.

Cognitive performance was assessed using various tests [12-17] to explore different aspects of cognitive functions, including attention, working memory, verbal memory, executive functioning, and processing speed (Table 1). The first author administered the tests after receiving training and supervision from a clinical psychologist. After completing the tests, scoring was done under the supervision of a clinical psychologist to improve reliability and reduce bias.

**Table 1:** Neuropsychological test to assess neurocognitive functions Citation: [17]

Test	Description	Scoring	Domain assessed
WAIS-IV Digit Symbol Substitution Test/Coding (DSST) [12]	The participant completes a series of accurately coded symbols. The higher the score, the better the performance.	Scoring was done by calculating the total number of symbols matched correctly within the time limit of 90 seconds.	Processing speed, visual-spatial abilities, and attention
Stroop Color-Word Test (SCWT) [13]	Participants are asked to identify the presented color words, printed hues, and control stimuli. Scoring is based on the number of correct answers given within the allocated time. The only Stroop scores used are Color Word (CW) and Interference scores.	Scoring was done by adding the total number of items correctly reproduced and the time in seconds.	Executive functions: Response inhibition (assess the ability to suppress cognitive interference)
F-A-S Test [14]	Participants have 60 seconds to list as many words as possible that begin with the letters F, A, and S.	Scoring was done by adding the total number of words produced by each letter within the given time limit.	Verbal fluency and selective attention
WAIS-IV Digit Span Test (DST) [12]	It includes three components: Digit Span Forward, Digit Span Backward, and Digit Span Sequencing.	Scoring was done by adding the longest sequence number repeated by the patients on digit span forward, backward, and letter-number sequencing. Higher scores suggest better performance.	Working memory and attention
Trail Making Test (TMT) [15]	It consists of two parts: A and B. Part A requires quickly connecting the numbers 1-25, which are randomly spread across a sheet of paper, in order. Part B is more complex, as it involves connecting numbers and letters in an alternating pattern (1-A-2-B-3-C, etc.).	The scores were calculated by noting the time it took to finish each task in seconds. Lower scores indicated better performance.	Perceptual speed and cognitive flexibility
Rey Auditory Verbal Learning Test (RAVLT) [16]	It consists of two distinct word lists (A and B). After presenting the lists, immediate recall is conducted, followed by repeated rehearsal. Delayed recall takes place after 20 minutes.	Scoring was done by adding the number of words recalled correctly on immediate recall, delayed recall (after 20 minutes), and errors.	Verbal learning and memory

All tests are validated for the Indian population [17] and were used in Hindi or English. After enrollment in the study, the participants received treatment (for the first time, they were being treated) as usual (detoxification/substitution) in accordance with standard protocol for treatment of opioid dependence using opioid agonists (Buprenorphine) by the Indian Psychiatric Society, 2019 [18]. As the participants consumed opium before visiting and were not in a withdrawal state, they were first recruited for the study. After getting the required information, the participants filled out questionnaires, and thereafter, the treatment was started only when they had withdrawal symptoms. Similarly, a sociodemographic profile was recorded for the controls, and a similar test battery was administered to assess cognitive functions.

Data were analyzed using the IBM SPSS Statistics for Windows, version 21.0 (released 2012, IBM Corp., Armonk, NY). Relationships between categorical variables were explored using the Chi-square test or Fisher’s exact test (for cells with fewer than five frequencies in more than 20% of cases). Continuous sociodemographic and clinical (cognitive) variables were compared using the Mann-Whitney U test due to the non-normal distribution of the data. Partial correlation was employed to obtain correlation coefficients while controlling for age, years of education, FTND, and FTND-ST score to investigate the association of cognitive functions with the level of exposure to natural opium, which is indirectly indicated by the age of opioid initiation, duration of opioid dependence, urine opioid ELISA level, and severity of opioid dependence on SOD-Q. A P-value under 0.05 was deemed statistically significant.

## Results

This study involved 26 participants from both groups. Table 2 outlines the sociodemographic and clinical profiles of the study participants. The comparison revealed no statistically significant differences between the two groups, including IQ scores. There was no difference in the frequency and severity of nicotine dependence between the groups. Among users, the average dose of Doda was approximately 1750 grams, while for Amal users, it was about 67 grams per month. No significant differences were noted between Doda and Amal users (13 each) in various sociodemographic and clinical parameters, including urine opioid levels (Table 3).

**Table 2:** Group comparison of sociodemographic and clinical variables n = number of participants; % = percentage; SD = standard Deviation; # = Fischer's exact; p = level of significance

Variables		Cases n (%)	Controls n (%)	χ^2^	p
Marital status	Single	5 (19.2)	4 (15.4)	-	1.000
	Married	21 (80.8)	22 (84.6)		
Occupation	Cleric/farmer/shop owner	10 (38.5)	15 (57.7)	2.526^#^	0.490
	Skilled	3 (11.5)	1 (3.8)		
	Semi/unskilled	10 (38.5)	7 (26.9)		
	Unemployed	3 (11.5)	3 (11.5)		
Family type	Nuclear	6 (23.1)	5 (19.2)	0.719	0.698
	Extended	11 (42.3)	9 (34.6)		
	Joint	9 (34.6)	12 (46.2)		
Locality	Urban	12 (46.2)	11 (42.3)	0.234^#^	1.000
	Rural	4 (15.4)	5 (19.2)		
	Town	10 (38.5)	10 (38.5)		
Presence of high-risk behavior		1 (3.8)	0 (0)	1.020^#^	1.000
History of nicotine use		20 (76.9)	18 (69.2)	0.391	0.532
Family history of substance use		21 (80.8)	20 (76.9)	0.115	0.734
		Mean (SD)	Mean (SD)	t	p
Age		36.0 (8.38)	32.96 (7.20)	1.437	0.157
Education (years)		12.80 (2.33)	13.46 (2.08)	-1.066	0.292
Income (Rupees/month)		19916.53 (10607.47)	20538.46 (11218.66)	-0.191	0.850
Intelligence Quotient (IQ) score		87.92	89.97	-1.130	0.264
Age of opioid initiation (years)		28.23 (7.00)			
Duration of opioid use (years)		7.84 (5.40)			
Duration of opioid dependence (years)		7.61 (5.15)			
Urine opioid ELISA level (ng/L)		0.306 (0.178)			
SODQ total score		38.5 (5.46)			
FTND score		4.14 (2.67)	2.66 (1.97)	1.116	0.288
FTND ST score		4.07 (1.68)	3.23 (1.36)	1.418	0.169

**Table 3:** Comparison of clinical variables among cases (Doda users (n = 13) versus Amal users (n = 13)) SD = standard deviation, t = t-value, p = level of significance, * p < 0.05

Variable	Doda users Mean (SD)	Amal users Mean (SD)	t	p
Duration of opioid dependence	9.54 (5.71)	5.70 (3.84)	2.016	0.055
Age (years)	38.54 (7.97)	33.62 (8.35)	1.537	0.137
Education (years)	12.84 (1.99)	12.77 (2.71)	0.082	0.935
Age of opioid initiation (years)	28.77 (7.33)	27.69 (6.90)	0.385	0.703
Duration of opioid use (years)	9.77 (6.08)	5.92 (3.98)	1.906	0.069
Usual monthly dose (gram)	1750 (629.15)	67.30 (36.66)	9.627	<0.001*
Urine opioid by ELISA	0.293 (0.168)	0.319 (0.193)	-0.368	0.716

The groups differed significantly across various cognitive domains. The cases scored significantly lower on the Stroop Color-Word test (p < 0.001) and higher on the Stroop Interference scores (p = 0.025). In addition, the cases scored significantly lower on the DST (sequencing) (p = 0.005). Significant intergroup differences were observed in TMT-B duration (p = 0.001), with cases taking longer to complete the task. Although no difference was found in the delayed recall trial of RAVLT, the performance of cases was poor, given their lower scores on RAVLT Hit (p = 0.003) and higher omission error scores (p = 0.004) (Table 4).

**Table 4:** Group comparison of cognitive variables FTND = Fagerstrom Test for Nicotine Dependence; FTND-ST = Fagerstrom Test for Nicotine Dependence – Smokeless Tobacco; DSST = Digit Symbol Substitution Test; DST = Digit Span Test; TMT = Trail Making Test; RAVLT = Rey Auditory Verbal Learning Test; SD = standard deviation; t = paired t-test; # = Mann–Whitney U test; p = level of significance; * = level of significance less than 0.05

Variables	Cases Mean (SD)	Controls Mean (SD)	t / U	p	Effect size	Confidence interval
DSST						
Coding	40.42 (6.48)	43.07 (4.50)	-1.714	0.093	-.475	-1.025 to 0.078
Error	0.500 (1.03)	0.50 (0.81)	359^#^	0.636	0.0656	-.544 to .544
Stroop Test						
Word	71.03 (14.13)	74.96 (9.13)	-1.189	0.240	-.330	-.876 to .219
Color	45.23 (11.09)	50.23 (7.45)	422^#^	0.123	0.2136	-1.080 to .027
Color-Word	24.31 (6.49)	32.11 (5.83)	-4.564	<0.001*	-1.266	-1.858 to -.663
Interference	21.04 (5.77)	18.04 (3.28)	2.304	0.025*	.639	.078 to 1.194
FAS Test						
Phonemic F	7.92 (1.69)	8.69 (2.03)	-1.481	0.145	-.411	-.958 to .141
Phonemic A	6.85 (1.87)	7.54 (2.48)	-1.135	0.262	-.315	-.860 to .234
Phonemic S	7.07 (1.38)	7.27 (1.61)	342^#^	0.933	0.0116	-.671 to .417
DST						
Forward	7.31 (1.22)	7.50 (1.10)	371^#^	0.531	0.0868	-.709 to .381
Backward	6.50 (1.10)	6.61 (.89)	355^#^	0.744	0.0453	-.658 to .430
Sequence	4.96 (0.77)	5.73 (1.00)	479^#^	0.005*	0.3897	-1.424 to -.286
Total	18.77 (2.66)	19.84 (2.18)	-1.596	0.117	-.443	-.991 to .110
TMT						
A- duration	30.69 (6.28)	28.77 (5.51)	1.172	0.247	.325	-.224 to .871
A - Error	0 (0)	0 (0)	0	0	0	0
B - Duration	77.53 (26.07)	57.16 (11.76)	3.631	0.001*	1.007	.425 to 1.581
B - Error	0.96 (1.95)	.84 (1.56)	345^#^	0.873	0.0222	-.479 to .609
RAVLT						
DR	9.27 (2.14)	10.04 (1.63)	-1.453	0.152	-.403	-.950 to .148
Hit	10.58 (1.39)	11.77 (1.36)	-3.119	0.003*	-.865	-1.431 to -.292
Omission	4.38 (1.41)	3.23 (1.36)	2.990	0.004*	.829	.258 to 1.393
Commission	2.77 (1.27)	2.77 (1.27)	338^#^	1.000	0	-.544 to .544

Table 5 presents the findings on partial correlation: urinary opioid levels positively correlated with Stroop-CW performance (p = 0.011). By contrast, the duration of opioid dependence showed a significant negative correlation with Stroop interference (p = 0.023) and DST sequence (p = 0.030). Several other tests indicated small effect sizes for correlation but did not reach significance. After applying Bonferroni corrections for multiple comparisons, none of the variables were found statistically significant, as the revised statistically significant p-value obtained was 0.002.

**Table 5:** Partial correlation of clinical and cognitive variables among the patients (n = 26) Partial correlation: controlled variables – age, education years, FTND, FTND-ST SODQ = Severity of Opioid Dependence Questionnaire; FTND = Fagerstrom Test for Nicotine Dependence; FTND-ST = Fagerstrom Test for Nicotine Dependence – Smokeless Tobacco; DSST = Digit Symbol Substitution Test; DST = Digit Span Test; TMT = Trail Making Test; RAVLT = Rey Auditory Verbal Learning Test;  * p-value <0.05

Variables	Age of opioid initiation	Duration of opioid dependence	Urine opioid level on ELISA	SODQ total
	Correlation coefficient (effect size)			
SODQ total	-0.255 (0.0650)	0.298 (0.0888)	0.404 (0.1632)	1.000 (1)
DSST coding	0.084 (0.0071)	-0.132 (0.0174)	-0.233 (0.0549)	-0.322 (0.1037)
DSST coding error	0.008 (0.0001)	-0.018 (0.0003)	-0.137 (0.0188)	-0.405 (0.1640)
Stroop W	0.210 (0.0441)	-0.274 (0.0756)	0.361 (0.1303)	-0.160 (0.0256)
Stroop C	0.294 (0.0864)	-0.363 (0.1318)	0.348 (0.1211)	0.088 (0.0077)
Stroop CW	0.087 (0.0076)	-0.150 (0.0225)	0.533* (0.2841)	0.226 (0.0511)
Stroop interference	0.414 (0.1714)	-0.481* (0.2315)	0.111 (0.0123)	-0.079 (0.0062)
Phonemic F	0.068 (0.0046)	-0.091 (0.0083)	0.028 (0.0008)	-0.032 (0.0010)
Phonemic A	0.272 (0.0740)	-0.310 (0.0961)	0.198 (0.0392)	-0.041 (0.0017)
Phonemic S	0.352 (0.1239)	-0.310 (0.0961)	-0.121 (0.0146)	-0.033 (0.0011)
DST Forward	0.067 (0.0045)	-0.103 (0.0107)	0.283 (0.0801)	-0.229 (0.0524)
DST Backward	0.306 (0.0936)	-0.334 (0.1116)	0.135 (0.0182)	-0.242 (0.0586)
DST Sequence	0.417 (0.1739)	-0.464* (0.2153)	-0.074 (0.0055)	-0.217 (0.0471)
DST Total	0.312 (0.0973)	-0.358 (0.1285)	0.174 (0.0303)	-0.294 (0.0864)
TMT A duration	0.011 (0.0001)	0.000 (0.000)	-0.306 (0.0936)	-0.224 (0.0502)
TMT B duration	0.088 (0.0077)	-0.058 (0.0035)	-0.052 (0.0027)	0.052 (0.0027)
TMT B error	0.024 (0.0006)	0.007 (0.000049)	0.186 (0.0346)	0.284 (0.0807)
RAVLT DR	0.095 (0.0090)	-0.121 (0.0146)	-0.245 (0.0600)	-0.359 (0.1289)
RAVLT hit	0.247 (0.0610)	-0.299 (0.0894)	-0.061 (0.0037)	-0.013 (0.0002)
RAVLT omission	-0.205 (0.0420)	0.258 (0.0666)	0.075 (0.0056)	0.047 (0.0022)
RAVLT commission	-0.016 (0.0003)	0.049 (0.0024)	-0.092 (0.0085)	0.000 (0)

## Discussion

Many studies indicate cognitive and psychomotor impairments in long-term opioid users, including declines in working memory, reduced cognitive flexibility, and increased impulsivity [19-21]. This is one of the few studies among patients dependent on natural opium that examines the impact of natural opium on cognitive functions and their correlates.

The sociodemographic characteristics of patients in our study are largely similar to those found in natural opioid-related epidemiological studies conducted in India [22]. No significant differences in IQ scores were observed across groups, which could have acted as a confounding factor when examining the relationship between opium use and subsequent cognitive impairment.

Study results show that patients dependent on natural opium perform significantly worse on the Stroop Test Color-Word scores (CW), Interference scores, Digit Span Test (sequencing), Trail Making Test Part B duration, and RAVLT errors of hits and omissions. Results suggest that natural opium use significantly affects cognitive domains such as response inhibition, cognitive flexibility, working memory, complex attention, and verbal learning.

Existing literature on cognitive functions among natural opium users is very limited. Similar to our study, a study from Iran found possible impairments in working memory and processing speed associated with natural opioid dependence [23]. This study revealed no difference between patients with OUD in the “oral/inhalation” or “oral only” groups; however, in the current study, all participants were oral users. By contrast, a recently published Indian study demonstrated that neuropsychological functions are relatively intact among patients dependent on natural opioids, with the natural opioid users differing from healthy controls only in the index of conceptual ability. Patients dependent on pharmaceutical opioids and heroin exhibited worse cognitive functions (verbal and visual working memory and response inhibition) than those dependent on natural opioids and controls, as measured by verbal and visual NBTs [4].

Most studies on cognitive functioning have been conducted with mixed opioid users. Among chronic opioid users, a meta-analysis suggests that robust impairments in verbal working memory, cognitive impulsivity, and cognitive flexibility have been observed, similar to our study findings [4]. In contrast to our findings, a meta-analysis concluded that the pattern of neuropsychological performance among patients with OUD appears to reflect mild generalized cognitive dysfunction, with a significant effect on complex psychomotor abilities [7].

In our study, we did not find a statistically significant difference in the TMT-A, DST Forward and Backward, Digit Symbol Substitution Test, F-A-S phonemic verbal fluency test, and RAVLT delayed recall. This suggests that perceptual speed, basic attention, processing speed, visuospatial abilities, verbal fluency, and delayed recall are relatively preserved in natural opium users. Similar findings were reported among natural opium users, where most cognitive functions do not differ significantly from healthy controls, except for conceptual ability [4]. By contrast, processing speed was the most affected domain, along with working memory, in a study of natural opioid users from Iran [23].

One methodological challenge when researching neuropsychological impairments linked to chronic opioid use is the influence of the chronicity and severity of dependence. The duration and severity of dependence can be considered proxy measures of cumulative exposure to the substance. Some investigators have found a direct relationship between both factors and the degree of cognitive decline in opioid users [24]. However, an inconsistent connection between the degree and duration of drug use and the results of neuropsychological tests is also reported [25].

Our study found that a longer duration of opioid dependence was linked to poorer response inhibition, mental flexibility, and working memory. In a previous study, Digit Backward Test (DBT) and DSST scores were correlated with duration of use, suggesting impairments in working memory, attention, processing speed, and visuoperceptual functions [22]. The literature presents inconsistencies regarding this association [19,26].

The varying quantities of opioids consumed may impact cognitive functions in different ways. In a previous study, opium use was quantified as the mean daily amount of opium usage (based on participants' self-reports and estimated in grams) multiplied by the duration of use [23]. Since Doda and Amal have different potencies, a standardized dose conversion has not been established. Therefore, urine quantitative analysis of opioids can provide consistent data regarding opioid exposure, which was assessed in our study. However, a significant correlation was found only with Stroop CW, suggesting that the greater the amount of opioids consumed, the worse the executive functions, such as response inhibition and mental flexibility. By contrast, in a previous study among patients dependent on natural opioids, the DBT score was associated with the quantity of opium use, indicating dose-related impairment in working memory [23]. Earlier studies in patients with chronic heavy prescription opioid misuse revealed mild to moderate impairments in attention, memory, and language [27-29].

Different types of opioids, their potencies, doses, routes of administration, and presence of adulterants may affect the profile and severity of neurocognitive functions through their neuropathological alterations in the central nervous system. Findings from a systematic review suggest that, compared to active opioid use, both buprenorphine and methadone treatment are associated with better neurocognitive functioning. However, buprenorphine is linked to improved executive functioning, attention/working memory, and learning/memory [30]. An Indian study indicates that patients receiving buprenorphine maintenance therapy experienced significant cognitive impairment, though it was limited to fewer cognitive domains; the extent and severity of impairment were greatest in the group with active opioid dependence. The performance of the opioid-dependent group was significantly poorer in the TMT-A & B compared to the buprenorphine maintenance group [8]. Another Indian study found that the group dependent on pharmaceutical opioids (which included low-potency synthetic opioids such as tramadol, tapentadol, pentazocine, dextropropoxyphene, and buprenorphine injection) had higher error scores in NBTs, and the time to complete the Trail Making-A test was longer compared to the natural opioid group [4]. Studies have reported that treatment with naltrexone in abstinent opioid abusers may result in less cognitive impairment than treatment with buprenorphine [8,31]. Overall, medication-assisted treatment, as well as naltrexone-based treatment for OUD, is beneficial for neurocognitive functioning, but data for comparison with natural opioids remains limited.

Tobacco use may impact neurocognitive functioning; however, no significant difference was observed in FTND and FTND-ST scores between the study groups, nor was either significantly correlated with any of the cognitive variables.

The available literature on the neurobiology of cognitive impairment in OUD can explain our study results. A recent meta-analysis showed that the frontocerebellar and frontoinsular circuits are predominantly involved in brain circuits in OUD, responsible for executive function (cognitive flexibility), decision-making, processing speed, and emotional processing [7]. Other studies suggest that the possible mechanism of impairment in executive functions could result from alterations in the brain’s structural and functional connectivity, especially in the frontostriatal, orbitofrontal, and amygdala circuits [32].

Although the present study provides important insight into the cognitive effects of natural opium, it has some limitations, such as a small sample consisting solely of male patients from a single hospital and its design as a cross-sectional study (cause and effect relations can’t be established). Therefore, the results cannot be generalized. The impact of tea or coffee consumption and its effect on cognitive functions was not evaluated. The cognitive assessment was not blinded, and multiple comparisons were made, which could have influenced the study results. Despite these limitations, the findings are significant, as the study was adequately powered, had matched controls for comparison, and covariates were examined and controlled in the analysis. Furthermore, the assessments were conducted while patients had not yet started experiencing withdrawal symptoms, and an attempt was also made to quantify the levels.

The study aimed to generate data on this particular group, as anecdotal reports suggest that natural users view opium as advantageous. In addition, these patients may have different treatment needs compared to others with OUD. 

## Conclusions

Response inhibition, cognitive flexibility, and working memory are the most affected domains of cognitive functioning, suggesting that cognitive dysfunction is limited to specific cognitive functions rather than global among natural opium users. The urinary opioid levels and the duration of opioid dependence demonstrate a significant correlation with cognitive impairment. The results are preliminary and need replication before guiding clinical practice. Meanwhile, a brief cognitive assessment will be helpful even in a busy clinical setting and should be part of a comprehensive assessment. These exploratory findings require longitudinal and interventional studies in a larger community sample.
